# The gut microbiota modulates airway inflammation in allergic asthma through the gut–lung axis-related immune modulation: A review

**DOI:** 10.17305/bb.2024.11280

**Published:** 2024-10-26

**Authors:** Meng Zhang, Ziwen Qin, Chuanjun Huang, Bin Liang, Xiuqing Zhang, Weitao Sun

**Affiliations:** 1 Department of Gastroenterology, People’s Hospital Affiliated to Shandong First Medical University, Jinan, Shandong Province, China; 2 The First Clinical Medical College, Shandong University of Traditional Chinese Medicine, Jinan, Shandong Province, China; 3 Department of Respiratory and Critical Care Medicine, Shandong Provincial Hospital Affiliated to Shandong First Medical University, Jinan, Shandong Province, China; 4 Department of Radiology, Dongying City Dongying District People’s Hospital, Dongying, Shandong Province, China; 5 Department of Respiratory Medicine, Dongying City Dongying District People’s Hospital, Dongying, Shandong Province, China

**Keywords:** Asthma, gut–lung axis, gut microbiota, airway inflammation

## Abstract

The human gut microbiota is a vast and complex microbial community. According to statistics, the number of bacteria residing in the human intestinal tract is approximately ten times that of total human cells, with over 1000 different species. The interaction between the gut microbiota and various organ tissues plays a crucial role in the pathogenesis of local and systemic diseases, exerting a significant influence on disease progression. The relationship between the gut microbiota and intestinal diseases, along with its connection to the pulmonary immune environment and the development of lung diseases, is commonly referred to as the “gut–lung axis.” The incidence of bronchial asthma is rising globally. With ongoing research on gut microbiota, it is widely believed that intestinal microorganisms and their metabolic products directly or indirectly participate in the occurrence and development of asthma. Based on the gut–lung axis, this review examines recent research suggesting that the intestinal microbiota can influence the occurrence and progression of allergic asthma through the modulation of cytokine immune balance and mucosal integrity. Though the precise immune pathways or microbial species influencing asthma through the gut–lung axis are still under exploration, summarizing the immune modulation through the gut–lung axis in allergic asthma may provide insights for the clinical management of the condition.

## Introduction

Asthma is a multifaceted condition characterized by reversible airflow limitation and airway hyperresponsiveness (AHR), with varying clinical manifestations and types of airway inflammation. Its onset is influenced by genetic, environmental, infectious, and nutritional factors, as well as intricate gene–environment interactions [1]. The current global prevalence of asthma exceeds 300 million individuals, with a projected rise to 400 million by 2025 [2]. Reports indicate that approximately 250,000 deaths annually can be attributed to asthma [3]. Despite ongoing research efforts, the precise etiology of asthma remains incompletely understood [4].

The gut microbiota comprises approximately 1000 species of microorganisms, encompassing 6–10 major phyla and consisting of 3000–5000 species, with a total mass ranging from 1–2 kg. The predominant phyla include *Bacteroidetes*, *Firmicutes*, *Proteobacteria*, and *Actinobacteria* [5]. The gut microbiota performs various essential functions in the human body, including organismal development and pathogen resistance [6]. Importantly, it also influences and maintains homeostasis by modulating immune responses in both the gastrointestinal system and distant organs, thereby playing a pivotal role in preserving overall health and managing diseases [7].

The “gut–lung axis” refers to the intricate interplay, regulation, and mutual influence between the gastrointestinal tract and respiratory system, mediated by microbial metabolism and immune function [8]. Immune cells in the intestinal mucosal tissue constitute a vital component of the body’s immune system, accounting for approximately 80% of all immune cells. During early development, these cells gradually and systematically colonize the digestive tract, helping establish a stable microbial ecosystem that is essential for intestinal health [9]. Early life is a crucial period for the establishment and development of the gut microbiota and immune system. Dendritic cells in human fetuses play a vital role in effective immunity and tolerance by migrating to lymph nodes and responding to microbial ligands, which helps reduce intrauterine inflammation [10].

During pregnancy, a woman’s gut and vaginal microbiomes undergo alterations that facilitate enhanced fetal energy extraction from maternal blood circulation [11]. Hormonal changes during pregnancy also influence maternal gut function and bacterial composition. Specifically, Vatanen et al. [12] observed that elevated progesterone levels prolong gastrointestinal transit time, leading to the entry of maternal metabolites into the circulatory system from the gut. These metabolites not only provide nutrients to the fetus but also impact the development of fetal inflammatory response and immune function. Cells and metabolites from the maternal gut microbiota can promote the expression of microbial pattern recognition receptors and hematopoiesis in the thymus and bone marrow, both of which play roles in regulating immunity [13]. At birth, bacteria transmitted from the mother can accelerate the shift in infant T helper (Th) cell dominance from Th2 to Th1 and Th17 immune phenotypes, along with mature regulatory immune mechanisms, thereby reducing the risk of allergic diseases and asthma in children [14].

Numerous studies have supported the association between the gut–lung axis and asthma in both humans and mice. Intrinsic bronchus-associated lymphoid tissue (iBALT) and gut-associated lymphoid tissue are both components of mucosa-associated lymphoid tissue, sharing correlated morphology and functionality [15]. In adults, intestinal mucosal tissues harbor approximately 80% of activated B cells [16]. The pathogenesis of intestinal flora and asthma is highly intricate. The intestinal flora is widely recognized as playing a pivotal role in regulating adaptive immune homeostasis. Disruption of the balance in intestinal flora contributes to asthma by influencing T cell development and differentiation, compromising the integrity of the intestinal epithelium and mucosal homeostasis, and impacting mast cell homing, among other mechanisms (as summarized by Gao et al., 2021) [17]. Consequently, lung microbial dysbiosis may play a causative role in the pathogenesis of asthma. The gut microbiota may affect asthma development and severity through various factors, including genetics and environmental influences. While the impact of maternal gut microbiota on the genetic predisposition to childhood asthma has been discussed, environmental factors—especially diet—also play a significant role in affecting gut microbiota diversity and asthma susceptibility.

Early colonization of the gut microbiota during infancy is essential for resisting pathogen colonization, facilitating immune system development and maturation, and supporting host metabolic processes [18]. Previous studies have shown an inverse correlation between the relative abundance of *Spirochaetes* and the incidence of preschool asthma, while a positive association has been observed between *Clostridium difficile* abundance and the development of this respiratory condition within the first three months after birth [19]. In breastfed infants, intervention with the probiotic strain EVC001 has been shown to upregulate the metabolites indolelactate and *Bifidobacterium infantis*-derived indole-3-lactic acid (ILA), which in turn upregulates the immunoregulatory molecule galectin-1. Galectin-1, a key member of the galactoagglutinin family, has been found to inhibit Th2 and Th17 cell polarization and induce interferon-beta (IFN-β) expression in these infants [20]. Additionally, short-chain fatty acid (SCFA) metabolites, important components of gut metabolic products, have demonstrated the ability to mitigate airway inflammation in individuals with asthma [21].

This article aims to provide an in-depth analysis of the current understanding of the relationship between the microbiome and asthma, focusing on the mechanisms by which the gut–lung axis influences immune modulation in the pathogenesis and progression of asthma.

### The dysbiosis of gut microbiota and airway microbiota affects the development of asthma

#### The influence of the gut microbiome on asthma

The gut microbiome undergoes significant changes during the first three years of life, characterized by a lower diversity index and greater interindividual variability [22]. Over time, diversity gradually increases, and the microbiota composition shifts toward an adult-like profile. For example, a study by Stokholm et al. found that immature gut microbiome development during the first year of life is a critical factor that increases asthma risk. This finding was supported by analyses of microbial community types. Having older siblings was identified as the only significant factor influencing membership in the more mature partitioning around medoids (PAM) cluster 2 at age one year. Microbial transfer from older siblings may promote the maturation of gut composition and, through appropriate stimulation of the developing immune system, protect against asthma in susceptible children [23].

Additionally, a study using 16S rRNA sequencing in a U.S. birth cohort identified distinct neonatal gut microbiota (NGM) compositions associated with the relative risk (RR) of childhood atopy and asthma. The highest-risk group had an increased likelihood of developing asthma, with a lower relative abundance of beneficial bacteria such as *Bifidobacterium*, *Akkermansia*, and *Faecalibacterium*. This group also showed a higher relative abundance of fungi, particularly *Candida* and *Rhodotorula*, along with a fecal metabolome enriched in pro-inflammatory metabolites [24].

According to a TwoSampleMR (TSMR) analysis investigating the causal relationship between gut microbiota and allergic diseases, the genus *Holdemanella* was identified as a potential risk factor for asthma [25]. In a recent metabolomics-based study, findings indicated that children with asthma have significantly decreased levels of amino acids and butyric acid metabolites, as well as a reduced quantity of butyric acid-producing bacteria, such as *Faecalibacterium* and *Roseburia*, while *Clostridium* spp. increase. This increase correlates negatively with fecal amino acids and butyric acid levels. Additionally, a significant decrease in fecal butyric acid levels in children with asthma is associated with elevated levels of total serum immunoglobulin E (IgE) and mite-specific IgE [26]. Gut microbiota dysbiosis is characterized by a shift in the relative abundance of different microbial taxa, resulting in a decrease in beneficial probiotic species and an increase in pathogenic bacteria that modulate SCFAs like propionic acid, thereby promoting Th2 inflammation.

#### The influence of the respiratory microbiome on asthma

Microorganisms can establish long-term symbiotic relationships with their hosts, referred to as colonization, without inducing disease. However, when an imbalance (dysbiosis) occurs in the microbial community, this disrupted population can trigger inflammation or infection [6]. Compared to healthy individuals, asthma patients exhibit reduced bacterial diversity but an increased microbial abundance in their respiratory microbiome, both of which are positively associated with asthma severity [27]. Bacteria play a significant role in asthma initiation and exacerbation, with experimental evidence indicating that bacteria such as *Streptococcus pneumoniae*, *Haemophilus influenzae*, *Moraxella catarrhalis*, *Chlamydophila pneumoniae*, and *Mycoplasma pneumoniae* can impact asthma’s occurrence, severity, exacerbation, and treatment response [28].

However, microbiome composition varies across different asthma phenotypes. Goleva et al. found discernible differences in microbial communities between hormone-sensitive and hormone-resistant asthma in bronchoalveolar lavage fluid samples at the genus level. Notably, certain genera, such as *Haemophilus influenzae*, are expanded in hormone-resistant asthmatic airways. Pre-exposure of airway macrophages from individuals with asthma to *Haemophilus influenzae* uniquely found in hormone-resistant asthma can activate p38 mitogen-activated protein kinase (MAPK), enhance interleukin (IL)-8 production, and impair glucocorticoid response [29]. Microbiome composition also varies between asthmatic and non-asthmatic patients across different age groups, and is closely associated with predicted forced expiratory volume in one second (FEV1) % values [30].

There are overlapping pathological changes between gastrointestinal and respiratory diseases, and intestinal inflammation may progress to pulmonary inflammation [31]. This bidirectional disruption is associated with an increased incidence of respiratory diseases such as asthma [32]. Correspondingly, patients with chronic gastrointestinal diseases have a higher incidence of lung diseases [33] (Table 1).

**Table 1:** Bacteria related with gut-lung axis microbial dysbiosis and asthma

**Bacterial genus**	**Compartment**	**Subjects**	**Microbiota linked to asthma**
*Bifidobacterium*	Gastrointestinal	Infants and children at risk for asthma	Decrease abundance associated with risk for asthma
*Clostridium difficile*	Gastrointestinal	Asthmatic and healthy children	The colonization at 1 month associated with asthma risk at the age of 6 years
*Lactobacillus rhamnosus*–associated fecal products	Gastrointestinal	Infants at high risk for asthma	Promote expansion of T-regulatory cells and IL-10 production *in vivo*, promoting tolerance for asthma
*Faecalibacterium, Roseburia, Clostridium*	Gastrointestinal	Preschool age asthmatic and healthy children at risk for asthma	Decreased abundance of Faecalibacteria, Roseburia associated with risk for asthma, increased abundance of Clostridium in asthmatic children
*Rothia, Lachnospira, Veillonella, Faecalibacterium*	Gastrointestinal	Infants and children at risk for asthma	Decreased abundance associated with risk for asthma
*Lachnospira, Clostridium neonatale*	Gastrointestinal	Preschool age asthmatic and healthy children	Decreased relative abundance of Lachnospira, increased relative abundance of Clostridium neonatale in asthmatic children
*Dolosigranulum*	Nasopharyngeal	Infants and children at risk for asthma	Prevalence associated with lower risk of viral respiratory infections and asthma
*Corynebacterium*	Nasopharyngeal	Asthmatic and healthy adults	Corynebacterium negatively associated with eosinophilic lung inflammation
*Haemophilus*	Nasopharyngeal, respiratory	Infants and children at risk for asthma, and asthmatic and healthy adults	Increased abundance in early life associated with increased frequency of viral infections and likelihood of developing persistent wheeze. Asthmatic status associated with increased abundance of Proteobacteria, especially Haemophilus
*Moraxella*	Nasopharyngeal, respiratory	Asthmatic children, children with respiratory disease, and preschool children with severe wheeze	Increased abundance in early life associated with increased frequency of viral infections and likelihood of developing persistent wheeze
*Streptococcus clostridium*	Nasopharyngeal, respiratory	Infants and children at risk for asthma, and children with rhinitis or asthma	Increased abundance in early life associated with increased frequency of viral infections and likelihood of developing persistent wheeze
*Haemophilus parainfluenza*	Nasopharyngeal, respiratory	Asthmatic and healthy adults	Increased abundance of Haemophilus parainfluenza; associated with activation of TLR4, proinflammatory IL-8, inhibition of corticosteroid-related pathway
*Proteobacteria*	Nasopharyngeal, respiratory	Adults with severe asthma	Increased abundance of Proteobacteria; associated with Th17-related genes
*Proteobacteria* with *Haemophilus* and *Neisseria*	Nasopharyngeal, respiratory	Asthmatic and healthy adults	Increased abundance of in asthmatics; general lower bacterial diversity associated with high Th2-related lung inflammation
*Neisseria*	Respiratory	Asthmatic and healthy adults	Increased abundance associated with asthma in adults
*Veillonella*	Respiratory, gastrointestinal	Children at risk for Asthma	Gastrointestinal decreased abundance

### The gut microbiota modulates asthma development via the gut–lung axis

According to the principles of Traditional Chinese Medicine, a fundamental theory suggests an interrelation between the lungs and intestines. This gut–lung relationship is supported in various aspects of modern medicine. Firstly, from an embryological perspective, the lungs and trachea originate from the foregut, and the respiratory epithelium and glands differentiate from the endoderm [34]. Secondly, the mucosal systems of the lung and intestine are part of a common mucosal immune system, with mutual interactions between mucosae [35]. Additionally, both the lung and intestine have neuroendocrine functions, with secreted neurotransmitters, peptides, and cytokines potentially facilitating complex interactions between them. For example, a neurotransmitter called vasoactive intestinal peptide (VIP), secreted by both the lungs and colon, is widely distributed in the intestinal tract [36]. Despite its high concentration in the gastrointestinal tract, VIP also has strong bronchodilatory effects on airways [37].

**Figure 1.**
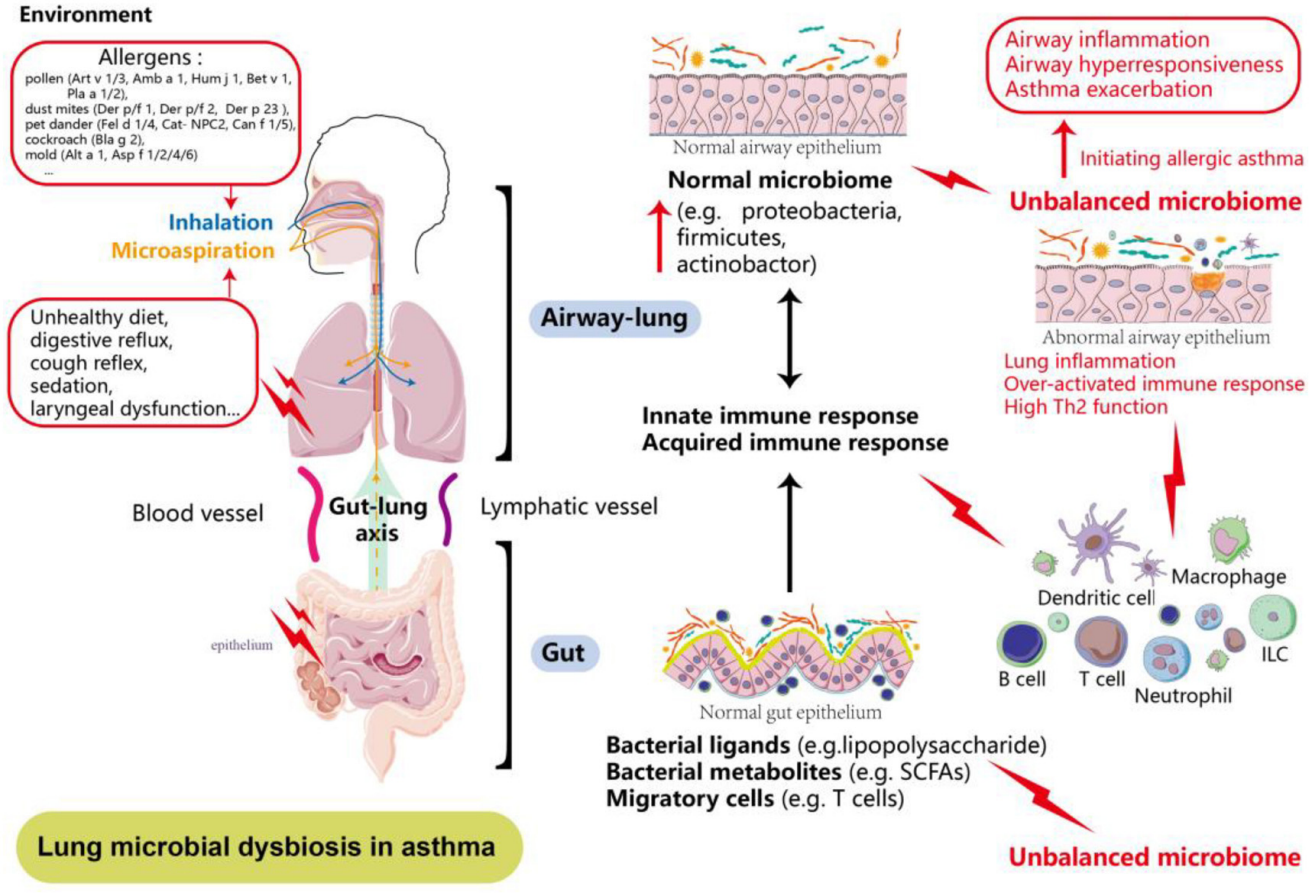
**Lung microbial dysbiosis in asthma: The gut–lung axis.** The lung microbiota plays a crucial role in maintaining a healthy immune response. Microbes in the oral cavity and upper airways shape the microbial communities in the lower airways and lungs. Environmental factors may contribute to lung dysbiosis, characterized by an increase in bacterial communities in asthma. Lung dysbiosis leads to heightened lung inflammation and immune dysfunction, initiating allergic asthma (illustrated in red). This inflammatory process may promote specific bacterial communities that contribute to further microbial dysbiosis.

Moreover, intestinal diseases can lead to significant toxin and bacterial proliferation in the bloodstream within the intestinal lumen [38]. Enterogenic endotoxins can accumulate and cause lung damage through circulation via blood vessels and lymphatic fluid [39]. In summary, the gut–lung axis may influence both physiological and pathological processes through complex immunomodulatory mechanisms, wherein gut microbiota and its metabolites impact respiratory immune responses (Figure 1).

#### Gut microbiota and their metabolites involved in asthma pathogenesis

##### Gut microflora directly affect the gut–lung axis

The microflora in the lung and intestine establish a direct connection within the gut–lung axis, enabling bidirectional regulation of function and state [8]. Lipopolysaccharides (LPS), a major component of the Gram-negative bacterial cell wall, elicit innate immune responses by activating Toll-like receptor (TLR) 4 on epithelial cell membranes and are closely associated with Th2-mediated allergic reactions in asthma [40]. The application of LPS to different organs in mice yields varying outcomes. For instance, when LPS is infused into the airways of mice, pulmonary dysbiosis occurs, accompanied by a significant increase in bacterial populations within 24 h in both the cecum and blood [41]. Conversely, studies have shown that mice with depleted intestinal flora due to antibiotic treatment have reduced resistance to influenza virus and *Escherichia coli*-induced pneumonia. However, intestinal administration of LPS in these mice significantly enhances their resistance, highlighting LPS’s role in augmenting airway immune response [42].

To further investigate the effect of LPS in asthma, a study using a dust mite-induced asthma mouse model found that gastrointestinal administration of LPS could inhibit Th2-high immune response, suggesting a preventive effect on asthma attacks [43].

##### Gut microbiota metabolites affect the gut–lung axis

Metabolites generated by the gut microbiota can have systemic effects on immune regulation across various respiratory and gastrointestinal disorders [44]. SCFAs, such as butyrate, propionate, acetate, and valerate, are primarily produced through the anaerobic fermentation of dietary fiber by cecal and colonic microflora and represent key microbial metabolites [45]. SCFAs act as immunomodulators by suppressing chemotaxis and adhesion of immune cells, inducing apoptosis in immune cells, and stimulating the secretion of anti-inflammatory cytokines [46]. Although SCFAs are barely detectable in lung tissue, they play a crucial role in protecting against allergic airway diseases [47].

Gut-derived SCFAs can reach the lungs and directly inhibit histone deacetylases (HDACs) through interaction with G protein-coupled receptors. Additionally, they enhance the differentiation and function of regulatory T (Treg) cells and Th1 cells by stimulating cluster of differentiation (CD) 4+ forkhead box protein P3 (Foxp3) [48]. In mice with allergic asthma, treatment with propionate and butyrate has been shown to reduce eosinophilic activity and suppress airway inflammation [49], suggesting that these SCFAs may promote the production of macrophages and dendritic progenitor cells, which migrate to the lungs and mature into CD11b+ dendritic cells, blocking allergen activation of Th2-effector cells [50]. This process regulates the proliferation of Th2 cells and reduces pro-inflammatory cytokine production.

In ovalbumin (OVA)-induced asthma mouse models, SCFAs have been shown to decrease levels of CD4+ T cells and IgE following vancomycin treatment, which results in alleviated AHR and reduced airway inflammation [51]. Additionally, mice fed a high-fiber diet exhibit increased blood SCFA levels and a lower incidence of allergic airway diseases, potentially due to SCFAs enhancing the function of CD8+ T cells [52].

The catabolism of cholesterol in the liver produces bile acids, which are subsequently modified by intestinal bacteria. These bile acids can influence the composition and structure of the gut microbiota [53]. In obese asthma patients, bile acids activate G protein-coupled receptors to enhance cyclic adenosine monophosphate levels, thereby suppressing the production of nuclear factor kappa-B (NF-κB)-mediated pro-inflammatory factors. This mechanism contributes to bronchial smooth muscle relaxation and alleviation of asthma symptoms [54]. A Canadian study found that infants at risk for asthma exhibited transient dysregulation of the gut microbiome within the first 100 days after birth. This dysregulation was associated with a specific asthma phenotype at one year of age and correlated with decreased urinary sulfate bile acid levels [55]. Furthermore, cesarean-section-born children, characterized by reduced levels of bile acids, tryptophan, and phenylalanine metabolites, were found to have an increased risk of asthma in school age [56].

In addition to SCFAs and bile acids, other bacterial metabolites also play roles in immune regulation in asthma. For example, tryptophan metabolites derived from diet and gut microbiota act as ligands for the aryl hydrocarbon receptor, contributing to immune modulation. Notably, allergic asthma patients exhibit significantly reduced serum levels of tryptophan metabolites [57]. In OVA-induced asthma mouse models, intraperitoneal injection of tryptophan metabolites alleviated asthma symptoms and reduced OVA-IgE and inflammatory markers [58]. These effects may be due to the regulation of Th17 and Treg cell differentiation [59]. Other intestinal flora metabolites, such as indole derivatives, polyamines, and deaminotyrosine, also have immunomodulatory effects [60–62]; however, their specific impacts on asthma require further investigation.

#### Asthma-associated immune response mediated by the gut microbiome

Contemporary asthma research posits a dynamic correlation between respiratory and intestinal microbiota, suggesting that the diverse and intricate mechanisms of the intestinal microbiota can modulate adaptive immunity, thereby influencing asthma-related immune responses and airway inflammation [8, 27]. These mechanisms involve the modulation of T cell development and differentiation, maintenance of intestinal epithelial integrity, and mucosal homeostasis, playing a pivotal role in the pathogenesis and progression of asthma.

##### Intervention in asthma by regulating Th1/Th2 balance

Th2-high inflammation is widely recognized as a crucial mechanism underlying allergic asthma [63]. Recent studies suggest that the gut microbiota may modulate asthma pathogenesis by regulating the balance between Th1 and Th2 responses [64, 65]. Mice exposed to OVA and raised in conventional housing conditions throughout early life exhibited enhanced intestinal flora diversity and attenuated airway inflammation associated with asthma, compared to mice housed under specific pathogen-free (SPF) conditions. This difference may be linked to the modulation of the Th1/Th2 balance [65]. The intestinal microbiota and its metabolites can influence intestinal lymphoid tissue, inducing a Th1 cell-mediated immune response to regulate the Th1/Th2 balance, thereby maintaining immune tolerance and preventing asthma development.

##### Intervention in asthma by regulating Th17/Treg balance

The Th17/Treg balance is another key mechanism in asthma pathogenesis [66, 67]. Th17 cells and their cytokines, such as IL-17, can induce the production of neutrophil chemokines in the airway epithelium, leading to neutrophil recruitment and inflammatory infiltration. Th17 cytokines can also trigger goblet cell hyperplasia and affect bronchial smooth muscle, resulting in increased mucus secretion and airway narrowing [68, 69]. Colonization by segmented filamentous bacteria in the murine intestine has been observed to elicit Th17 cell development, which is associated with increased expression of pro-inflammatory genes [70]. Treg cells, on the other hand, suppress excessive Th2 immune responses and are crucial for maintaining immune homeostasis in allergic conditions like asthma [71].

The intestinal microbiota plays a key role in regulating Th17/Treg cell differentiation and homeostasis, either through direct modulation of receptors and cytokines or through energy metabolism pathways [72]. Research has primarily focused on early life stages, where delayed maturation of the gut microbiota can induce Th17 expression and exacerbate allergen-driven AHR, worsening asthma severity [73]. Conversely, exposure to maternal gut flora and breastfeeding practices have been shown to promote a balanced Th17/Treg ratio in offspring, reducing asthma risk [74, 75]. Administration of bifidobacteria to breastfed infants suppresses Th2 and Th17 cytokines while promoting IFN-β production, thereby reducing airway inflammation [20].

Another study found that mice given oral *Lactobacillus* showed higher levels of Treg cells and Foxp3 mRNA, along with decreased levels of inflammatory cells like granulocytes, Th17, and Th2 cells, suggesting that intestinal flora can regulate the differentiation of naive CD4+ T cells into Th17 cells, thus maintaining the Th17/Treg balance and modulating the immune response [76]. Treg cells also influence IgA levels, which play an anti-infective role by modulating TGF-β production [77]. Studies have shown that a diverse microbiota is crucial for maintaining optimal IgA function, as evidenced by higher IgA levels in SPF mice compared to germ-free mice [78]. Low IgA binding to intestinal flora has been associated with increased asthma risk in children [79]. In murine models with intact immune function, Treg cells stimulate IgA secretion on mucosal surfaces, aiding in the expulsion of pathogenic ligands and reducing systemic inflammation [80]. Conversely, antibiotic-treated asthmatic mice, with significantly reduced Treg cell accumulation and elevated IgE levels, exhibit increased airway inflammation [81].

As previously mentioned, Treg function is modulated by SCFAs produced by the intestinal microbiota. Dietary SCFAs have been shown to reverse allergic inflammation in the lungs of mice [51]. Additionally, *Clostridium*-induced Tregs help maintain intestinal immune homeostasis [82], while polysaccharide A (PSA) can mediate the transformation of CD4+ T cells into IL-10-producing Foxp3+ Tregs and inhibit the Th17 response via TLR2 [83]. The intestinal microbiota also expresses outer membrane proteins that promote Treg development and functionality while inhibiting excessive Th2 cell activation, which is closely associated with asthma pathology [84]. In summary, the intestinal microbiota is crucial for maintaining immune homeostasis by balancing Th17/Treg responses and thereby reducing asthma progression.

##### Intervention in asthma by regulating mucosal immunity

Both the respiratory and digestive tracts have mucosal structures comprising epithelium and lamina propria, integral components of the shared immune mucosal system. The microbial communities inhabiting the lungs and intestines interact with host immunity, establishing a personalized micro-ecological environment [85]. *Clostridioides difficile* colonization in the intestine can trigger inflammatory responses, including TLR5 binding and NF-κB activation, which regulate the expression of pro-inflammatory genes, leading to mucosal inflammation and epithelial barrier disruption [86]. This highlights the importance of the intestinal epithelial barrier in immune regulation.

Intestinal flora can produce metabolites that reinforce the epithelial barrier, using the gut–lung axis as a bridge to support mucosal immune function and block pathways involved in airway remodeling, thereby influencing asthma development [87]. Studies show that the intestinal microbiota can protect mice from LPS-induced acute lung injury by regulating the TLR4/NF-κB signaling pathway [88, 89]. SCFAs, as primary metabolites of the intestinal microbiota, play a pivotal role in preserving intestinal epithelial integrity and mucosal homeostasis. Butyrate, an SCFA, promotes the development of the intestinal barrier by inducing tight junction protein formation through myosin light chain kinase and Rho kinase activity [90]. Additionally, gut-derived acetate, another SCFA, has been shown to protect airway tight junctions against influenza-induced lung injury, supporting the idea that SCFAs preserve airway epithelial integrity and mucosal homeostasis [91]. This suggests a novel approach for addressing asthma through gut–lung mucosal immunity.

##### Intervention in asthma by regulating other pathways

In addition to the above pathways, the gut microbiota modulates asthma progression through various other mechanisms. TLRs, a family of transmembrane protein receptors, are crucial for innate immune system activation. Early-life microbial alterations can impair TLR activation and increase susceptibility to allergic diseases like asthma [92]. In a mouse model, probiotic-mediated asthma protection required maternal TLR signaling [93]. Mice exposed to *Acinetobacter lwoffii* F78 exhibited enhanced resistance to airway allergies in their offspring, while impaired TLR function abolished this protective effect [74]. In TLR-deficient mice, the anti-inflammatory effects of various gut microbiomes were diminished [94], suggesting that TLR signaling modulates Treg activation, potentially influencing asthma susceptibility.

The NOD-like receptor thermal protein domain-associated protein 3 (NLRP3) inflammasome recruits inflammatory cells, contributing to immune responses in the lungs and gastrointestinal tract [95]. The NLRP3 inflammasome is closely associated with asthma, especially severe asthma, and its modulation by the gut microbiome may play a key role in asthma pathogenesis [96]. *Vibrio cholerae* in the intestine can activate IL-1β secretion via the NLRP3 inflammasome [97]. In germ-free mice, IL-1 induction is absent [98], while macrophage-stimulated fecal contents from normal mice elicit IL-1 production *in vitro*, highlighting the role of colonized bacteria in activating the NLRP3 inflammasome and promoting asthma progression [99].

Invariant natural killer T (iNKT) cells enhance the body’s responsiveness to allergens, promoting Th1/Th2 imbalance and asthma attacks [100]. Studies show a close association between iNKT cell expression and microbiota status. In asthmatic mice lacking intestinal flora, iNKT cell levels in the lungs are elevated, leading to pronounced airway inflammation. Reintroducing intestinal flora attenuates this response, with notable expression of chemokine Cxcl16 by iNKT cells, indicating a correlation among intestinal flora, iNKT cell expression, and asthma [101].

TGF-β1, a pro-fibrotic factor implicated in airway remodeling in asthma, exerts its effects through SMAD proteins, which mediate most TGF-β1 signaling [102]. Increasing bifidobacteria and lactobacilli in asthmatic rats has been shown to alleviate asthma symptoms by downregulating TGF-β1, SMAD4, and SMAD7 levels, while upregulating SMAD3 expression [103, 104].

In summary, gut microbiota metabolites, such as SCFAs, regulate T-helper cell balances (Th1/Th2/Th17), influencing cytokines like IL-17 and TGF-β to promote a healthy immune response. In the gut–lung axis, migration, interaction, and signaling of immune cells, as well as direct effects of gut metabolites, shape the immune environment of the airway, modulating cytokine production and impacting airway inflammation and AHR in asthma. Understanding the microbiota’s role in asthma-related inflammatory pathways opens opportunities for novel therapeutic strategies targeting microbial communities to treat and prevent asthma from a fresh perspective.

### Asthma prevention and treatment strategy based on the gut–lung axis theory

Currently, the clinical management of asthma predominantly relies on glucocorticoids and β2 receptor agonists, which can alleviate symptoms and slow disease progression within a specific timeframe. However, achieving long-term curative effects for asthma remains challenging. Studies have shown significant differences in the fecal microbiome of asthma patients with and without inhaled corticosteroid (ICS) treatment, suggesting that ICS use may impact gut dysbiosis and associated functional pathways [105]. However, it is still unclear whether ICS-induced changes in gut microbiota directly contribute to asthma severity. In one study, oral antibiotics were found to relieve allergic asthma in post-weaning mice [106], a result that contradicts the mainstream view that antibiotic exposure during early life or pregnancy worsens allergic airway inflammation by affecting gut microbiota and lung lipid metabolism [107, 108]. Although the impact of oral drugs on gut microbiota and allergic asthma outcomes is not yet fully understood, these findings suggest that gut microbiome interventions may hold potential in asthma treatment.

The discussion on the gut–lung axis mechanism implies that modulating the gut microbiota could be a novel therapeutic approach for managing pulmonary disorders. The relationship between allergic asthma and diet-driven microflora has been extensively studied. From a gut microbiota perspective, enhancing the diversity of intestinal flora and promoting SCFA production may offer benefits for asthma prevention and treatment. Oral agents, including dietary interventions, probiotics, and prebiotics, can be used to modify gut microbiota diversity and abundance, indirectly influencing respiratory microbiota [109]. Animal studies have shown that modulating the gut microbiota can significantly improve airway inflammation and hyperreactivity in asthma, relieving symptoms and preventing disease progression [110].

#### Probiotics

Probiotics are beneficial live microorganisms, primarily derived from healthy human gut flora, known for their immune-regulating, antioxidant, and antimicrobial properties [111]. Given the role of gut flora in atopic diseases, probiotics have emerged as a promising approach for asthma prevention and treatment. Numerous studies have shown that both single and combined probiotic strains can influence the maturation and tolerance of intestinal mucosa, affect dendritic cell development, and regulate systemic immune responses, ultimately yielding significant therapeutic effects on asthma [112].

*Lactobacillus*, recognized as the most promising probiotic for asthma prevention and treatment, has garnered extensive validation in animal studies. Administration of *Lactobacillus fermentum* to asthmatic mice significantly improved lung inflammation and fibrosis, accompanied by reduced levels of inflammatory mediators such as IL-4, IL-5, and IL-13 in lung tissue. Additionally, the expression of TLR2 and TLR4 proteins was notably downregulated [113]. A mixture of *Lactobacillus salivarius* and *Lactobacillus johnsonii* showed optimal therapeutic effects in OVA-induced allergic asthma mice, mediated by FOXP3 and Treg induction via gut microbiota and lung accumulation [114]. Similarly, probiotics administered to asthma patients increased FOXP3 and Treg levels while suppressing the Th17-mediated inflammatory response, leading to improved asthma symptoms [115].

*Lactobacillus rhamnosus* (LGG) has also been shown to reduce airway resistance in mice and lower levels of IgE, inflammatory cells, and Th2 cytokines in serum and bronchoalveolar lavage fluid [116]. Supplementing *Lactobacillus rhamnosus* before sensitization reduced hyperresponsiveness to acetylcholine in mice, helping to prevent asthma [117, 118]. In children with asthma and allergic rhinitis, treatment with *Lactobacillus gasseri* significantly improved allergic symptoms and reduced inflammatory factor expression [119]. In addition to *Lactobacillus*, other probiotics such as *Bifidobacterium*, *Streptococcus*, and *Propionibacterium* are commonly used to support overall health [120]. Complex probiotic formulations, such as *Bifidobacterium longum* BB536, *Bifidobacterium infantis* M-63, and *Bifidobacterium breve* M-16V, have shown significant efficacy in alleviating asthma symptoms [121].

While oral administration is the standard route for probiotics, intranasal administration of *Lactobacillus paracasei* has shown greater effectiveness in female asthmatic mice [122]. Conversely, nasal administration of *Lactobacillus rhamnosus* in male mice appeared less effective [123, 124]. Moreover, there is no consensus on the optimal dosage of probiotics for asthma treatment [114]. Therefore, while probiotics show promise in asthma management, more research is needed to establish the best administration routes and dosages for effective treatment [125].

#### Dietary fiber

Dietary fiber supplementation is a crucial strategy for asthma prevention by modulating the microbiome. As a multifaceted dietary component, it exerts preventive effects on asthma by enhancing the epithelial barrier, promoting Treg cell induction, mitigating Th2 polarization, and inhibiting excessive mast cell secretion [126]. Studies in asthmatic mice treated with dietary fiber have shown reduced eosinophilic airway inflammation, lower IgE levels, and diminished Th2-related inflammatory mediators, leading to significant improvement in asthma symptoms [127]. Furthermore, dietary fiber interventions have been found to induce prenatal epigenetic gene imprinting through immune regulation, evidenced by a reduction in acute allergic reactions in sensitized maternal mouse offspring exposed to allergens [110]. Research on adult asthma patients has demonstrated a negative association between dietary fiber intake and increased airway eosinophils, alongside improved lung function [128]. These findings suggest that higher dietary fiber consumption may contribute to a decreased likelihood of developing asthma within the population, while also potentially mitigating symptoms such as coughing, wheezing, and sputum production [129].

The approach to asthma prevention through intestinal microbiota modulation is still in the research stage. Various single or complex agents involving dietary nutrition, probiotics, and prebiotics are being explored, though there remain controversies regarding their universality, effectiveness, and appropriate modes and dosages. Therefore, further investigation is necessary.

## Conclusion

The gut–lung axis has become a research focus in diseases such as asthma. With advancements in omics technology, numerous clinical and animal studies targeting intestinal flora for asthma treatment continue to emerge, providing new insights into asthma treatment and prevention beyond conventional approaches. However, further investigation is needed to deepen our understanding of the underlying mechanisms of the gut–lung axis, and additional robust experimental evidence is essential to substantiate hypotheses regarding its pathways.

Firstly, the complexity of the human intestinal ecosystem poses challenges in replicating phenomena observed in *in vitro* and *in vivo* animal studies within clinical contexts, complicating efforts to unravel the mechanisms governing lung-intestine interactions. Secondly, limited research examines how microbiota alterations impact distant organs through host modulation, underscoring the need for more exploration into the interplay of mucosal immunity and neuroendocrine communication between the lung and intestine.

Although the mechanisms underlying the gut–lung axis are not yet fully understood, it represents a promising avenue for asthma prevention and treatment. Numerous clinical and animal studies have targeted the intestinal microbiota to alleviate asthma symptoms or reduce asthma risk, with encouraging results that support further investigation into the gut–lung axis. However, barriers to translating microbiota research into clinical therapies—such as patient variability, microbial resilience, and regulatory challenges—must be addressed as the field progresses. Emerging concepts, such as personalized medicine based on an individual’s microbiome, could lead to more tailored approaches to asthma treatment. Additionally, research areas like synthetic biology and microbiome engineering may offer future-oriented strategies for developing microbiota-based therapies against asthma.

In summary, harnessing the gut microbiota could pave the way for groundbreaking advances in asthma research and therapeutic interventions.
